# Clinical Knowledge Mining Based on Image Enhancement Algorithm: Endoscopic Clinical Analysis of Peptic Ulcer in Children

**DOI:** 10.1155/2022/3716829

**Published:** 2022-07-01

**Authors:** Lina Qiao, Yarui Zhou, Ying Shen, Qi Sun

**Affiliations:** Department of Pediatric, Affiliated Hospital of Weifang Medical University, Weifang 261041, Shandong, China

## Abstract

The incidence rate of peptic ulcer is increasing gradually. Medical images can meet the needs of patients as an auxiliary diagnosis and treatment method for peptic ulcer. However, in the long-term treatment, the actual effect is average, and the diagnosis effect of gastrointestinal diseases will gradually deteriorate. In this paper, we use an image enhancement algorithm to study the mechanism of peptic ulcer from the perspective of a medical image. In this paper, 56 images of children with peptic ulcer were selected, and the gastroscopy based on the image enhancement algorithm provided technical support for the rapid diagnosis of patients with peptic ulcer. Experimental results show that the clinical features of peptic ulcer have different characteristics according to the age difference of patients, which can play a positive role in promoting the treatment of patients of different ages.

## 1. Introduction

The early image enhancement algorithm based on nonlearning is to modify the dark image directly to make the processed image have higher contrast, to achieve the purpose of dark image enhancement. The commonly used histogram equalization (he) technology can make the histogram of the image as balanced as possible, to achieve the purpose of image brightness as a whole. In addition, abnormal energy homeostasis may also lead to comorbidity of T2DM and AD.

*Helicobacter pylori* is the most important cause of peptic ulcer disease. Ji and Sang considered it important to exclude false-negative results in *Helicobacter pylori*-negative peptic ulcer and that long-term maintenance pharmacotherapy should be considered due to the risk of recurrence and the high risk of disease-related complications [1]. Lind et al. used pooled data from both studies for two-way and multifactorial analyses, adjusted for demographics, and established risk factors for peptic ulcer and peptic ulcer bleeding, and disease severity variables [2]. Medical treatment has been widely used for peptic ulcer disease, but perforation is a serious complication. The rationale for the Kocakusak's study was to assess the effects of Islamic Ramadan fasting on perforation of peptic ulcers [3]. Sgourakis designed to evaluate studies of endoscopic hemostasis at low doses versus high doses of proton pump inhibitors (PPIs), including newly published randomized controlled trials (RCTs) and to conclude whether low doses of PPIs can produce comparable results with high doses of PPIs [4]. Over the past few decades, the incidence has decreased in patients with perforated peptic ulcer (PPU). At the same time, more and more surgeons are adopting laparoscopic treatment for this condition. The objective of the Beuran and Lica's study was to assess the early postoperative outcomes of laparoscopic treatment of perforated peptic ulcers performed at eight Romanian surgical centers with extensive experience with laparoscopic surgery [5].

In the image feature enhancement algorithm, Land E improves the contrast and brightness by increasing the brightness of the dark area and reducing the brightness of the bright area, expands the dark area, and compresses the brightness at the same time. Based on a defogging algorithm and the Retinex model method, the dark image can also be added [6]. Land and Mc Cann proposed the Retinex theory. When it is used to explain the color perception characteristics of the human visual system, it is assumed that the image is decomposed into reflection and illumination. Thus, two traditional methods are developed: single-scale Retinex (SSR) limits the smooth illumination mapping by the Gaussian filter, and multiscale Retinex (MSRCR) expands the SSR of the multiscale Gaussian filter and color restoration [7]. At present, Zhang et al. have designed a depth network based on Retinex theory, retirement, which integrates image decomposition and illumination mapping, uses the BM3D (block-matching and 3D filtering) algorithm to remove the noise influence of reflection components, and then reconstructs the image [8]. However, retirement cannot estimate the illumination correctly, which leads to color distortion. At the same time, due to the use of BM3D denoising, image details will be lost. In this regard, Lu et al. improved the retirement algorithm, using the dark gene image enhancement network (CRNET), to solve the problem of image color distortion and detail loss by adding channel attention structure (CA) to the network to enhance image output and adding image gradient loss function and image detail enhancement module to enhance image detail [9]. Zotin makes the proportion of positive and negative samples in the data set reach the balance after generating the *anti*-network amplification method and carries out the gene image classification experiment [10]. However, data show that metformin may increase the risk of AD. The above studies only analyze the treatment of colorectal cancer from the perspective of medical images and nutritional status of patients, and lack of research on the genetic mechanism. Colorectal cancer is one of the common malignancies, which consists of colon and rectal cancers. The incidence of colorectal cancer from high to low is the rectum, sigmoid colon, cecum, ascending colon, descending colon, and transverse colon, which in recent years has a trend toward proximal (right colon) development. Its onset is closely related to lifestyle, genetics, and colorectal adenoma.

In this paper, we use an image enhancement algorithm to study the mechanism of peptic ulcer from the perspective of a medical image. Gastroscopic imaging is used for clinical typing and medical diagnosis according to different ages from the perspective of medical images.

## 2. Experiments and Methods

### 2.1. Research Content

In this paper, gastroscopic images of children with peptic ulcers in our hospital were selected as training and experimental objects. According to the clinical manifestations of patients, patients are divided into primary acute ulcer group, subacute ulcer group, chronic ulcer group, and stress ulcer group, a total of 4 groups. Then, we compared the enhancement effects of different algorithms on the image and compared it with the original image.

### 2.2. Image Enhancement Algorithm

As a kind of super wavelet, the image enhancement algorithm based on retirement network of nonsubsampled contourlet transform has many good properties, such as achieving the best sparse representation of linear singularity, decomposing the image in different directions with all kinds of anisotropy, and eliminating the pseudo-Gibbs phenomenon with translation invariance, so NSCT is widely used in many fields of image processing. Based on the analysis of the statistical characteristics of NSCT-domain coefficients, this paper proposes a method to enhance the side-scan sonar image. Experiments show that this method not only eliminates most of the speckle noise and suppresses the trivial texture but also improves the gray contrast of the low-frequency region and enhances the edge details of the high-frequency region [11]. In this paper, nine side-scan sonar images are randomly selected and denoised according to the image enhancement algorithm. The denoising indexes of each method are calculated and the denoising effect is analyzed.

### 2.3. Ulcer Feature Recognition Algorithm Based on Image Enhancement

In this paper, we use neural network image enhancement algorithm to improve the recognition rate of colorectal cancer patients' medical images. From the abscissa of the peak value to the right along the direction of the increase of the difference in coefficient to the position of the maximum value *Z*_*M*_, we get

(1) $$\begin{array}{c} Z_{m}=\left\{P_{1}|D,L,f_{2}\frac{P_{3}|N,M,I}{P_{2}|f_{1},\mu }\right\},\\ P\frac{Z_{m}}{Q}=\overset{U}{\underset{S-1}{\sum }}\overset{K}{\underset{d-1}{\sum }}f_{s},D+N_{s},d. \end{array}$$

The interval statistical distribution *P* contains three morphological components: texture, weak edge, and strong edge. Because the difference between the maximum and minimum direction subband coefficients in the same scale of NSCT domain of texture, soft edge, and strong edge increases in turn, and the sparsity also increases gradually, it can be inferred that texture, weak edge, and strong edge have the same characteristics. The strong edge is distributed in the right region of the peak in the order of difference in coefficient from small to large [12]. Therefore, the average value *A*_*K*_ of the *k* channel descriptor value is

(2) $$\begin{array}{c} A_{k}=\frac{\sum_{i=1}^{k}\sum_{q\in D_{i}}{\left|m-n_{i}\right|}^{2}}{U},\\ A=f\left(x\right)=\frac{\sum_{j\subset Q}c_{j}x_{j}/\sigma \left(X_{j}^{\prime }\right)-p}{C*M}, \end{array}$$

where *u* is the characteristic graph before global average pooling, (*m* − *n*) is the global pooling function, and *K* is the value of the *k* characteristic I at position (*m*, *n*). The final channel statistic *s* is obtained by taking the reduction factor *R* as the scale to do downsampling, and then multiplying it by *T* (taking *r* as the scale to do upsampling)

(3) $$\begin{array}{c} S_{1}=\left\{R_{1}\left(t-4\;\Delta t\right),R_{1}\left(t-3\;\Delta t\right),R_{1}\left(t-2\;\Delta t\right),R_{1}\left(t-\Delta t\right)\right\},\\ F\left(s\right)=2n\ln\left(s\right)+n\ln\left(s\right)+n\left\{\frac{n+tr\left(R\right)}{n-2-tr\left(R\right)}\right\}, \end{array}$$

where *F* is sigmoid function, LN is *R* function, and tr () is filter of convolution layer. The statistic *s* is used to readjust the input *Q*:

(4) $$\begin{array}{c} Q\left(\delta \right)=R\left(y\left(T-1\right),u\left(T-d-1\right)\right),\\ \Delta T^{l3}=-\eta \delta^{l3}Q^{l3}\\ =\eta \left(t-y\right)f^{\prime }\left(X^{l3}T^{l3}\right)Q^{l3}. \end{array}$$

After adding the channel attention layer, the original feature information will become (*T*, *T* + 1) times and *R* is the weight of the *T* layer after adding the channel attention layer {}

(5) $$\begin{array}{c} R_{2}=\left\{R_{2}\left(t-4\;\Delta t\right),R_{2}\left(t-3\;\Delta t\right),T_{2}\left(t-2\;\Delta t\right),T_{2}\left(t-\Delta t\right)\right\},\\ T_{i}=R\left(u_{i},v_{i}\right)+\overset{p}{\underset{j=1}{\sum }}R_{j}\left(u_{i},v_{i}\right)x_{ij}+\varepsilon_{j}\beta_{j}. \end{array}$$

In order to preserve the image details, an image gradient loss function is added. The gradient of *X* and *Y* axis of an image is calculated by two-dimensional convolution

(6) $$\begin{array}{c} \Delta TG^{l}=-\eta \frac{\partial X}{\partial Y^{l}}\\ =\eta {\left(X^{l}\right)}^{T}\delta^{l},\\ \log_{2}n+1\leq TG\leq \log_{2}n^{2}. \end{array}$$

TG is the gradient element and *Z* is the gradient intensity:

(7) $$\begin{array}{c} Z\left(d_{i},w_{j}\right)=P\left(d_{i}\right)P\left(w_{j}|d_{i}\right),\\ Z\left(Ai,Aj\right)=\left[\frac{\log\left(\left|x_{A_{i}}-a_{A_{j}}\right|/w_{A_{j}}\right)}{\log\left(w_{A_{i}}/w_{A_{j}}\right),}+\frac{\log\left(\left|y_{A_{i}}-y_{A_{j}}\right|/h_{A_{j}}\right)}{\log\left(h_{A_{i}}/h_{A_{j}}\right)}\right], \end{array}$$

where *x* and *y* represent the spatial position and *a* is the element index. The frequency of gradient image is

(8) $$\begin{array}{c} Z_{R}^{A_{i}}=H_{G}^{A_{i}A_{j}}\cdot W_{A},\\ w_{G}=\min\left\{0,W_{G}\cdot \varepsilon \left(f_{G}^{A_{i}},f_{G}^{A_{j}}\right)\right\}. \end{array}$$

The loss function is *Z*_1_

(9) $$\begin{array}{c} \Delta Z_{1}^{l2}=-\eta \delta^{l2}w^{l2}\\ =\eta \delta^{l3}{\left(Z^{l3}\right)}^{T}f^{\prime }\left(X^{l2}Z^{l2}\right)X^{l2},\\ Z=N\left(d_{i},w_{j}\right)\\ =\frac{Z\left(w_{j}|d_{i}\right)}{f^{\prime }\left(X^{l2}Z^{l2}\right)X^{l2}}, \end{array}$$

where *W* is 1 norm and *Z* and *X* are input and output image gradients, respectively. The loss function of network decomposition and reconstruction is based on the loss function of retinexnet algorithm. Suppose that both low *R* and normal *R* can reconstruct the image from the corresponding illumination map, so the reconstruction loss *W* is

(10) $$\begin{array}{c} W^{l2}=\delta^{l3}{\left(Z^{l3}\right)}^{T}f^{\prime }\left(X^{l2}Z^{l2}\right),\\ W=\Delta Z^{l}\\ =-\eta \frac{\partial X}{\partial W^{l}}\\ =\eta {\left(X^{l}\right)}^{T}\delta^{l}+C,\\ \Delta M_{i}=M_{j}-\eta E{}^{\prime }\\ =\eta x_{i}\left(t-y\right)f^{\prime }\left(Z\right)\\ =\eta x_{i}E. \end{array}$$

Constant reflectivity loss *R* is introduced to constrain the consistency of reflectivity, *C* is constant, and *e* is positive correlation. The decomposition process of control gene characteristic map, reflection map, and illumination map is as follows

(11) $$\begin{array}{c} Z=\frac{n}{Q_{\text{ikjl}}}\sqrt{\overset{n}{\underset{s=1}{\sum }}{\left(x_{ik}\left(\varepsilon \right)-x_{jl}\left(\varepsilon \right)\right)}^{2}Q_{\text{ikjl}}\left(\varepsilon \right)},\;Q_{\text{ikjl}}>0,\\ R=\frac{Q}{Z}-\frac{\left(2\mu_{x}+Q_{1}\right)\left(2\sigma_{xy}+Q_{2}\right)}{\left(\mu_{x}^{2}+Q_{1}\right)\left(\sigma_{x}^{2}+Q_{2}\right)}. \end{array}$$

The total loss function *Q* is

(12) $$\begin{array}{c} Q=\left(t-y\right)f^{\prime }\left(X^{L}W^{L}\right)+E,\\ \frac{\partial Q}{\partial W^{l}}=-\;{\left(x^{l}\right)}^{T}\delta^{l}+\frac{Z}{R}, \end{array}$$

where *Z*, *R*, and *W* are the corresponding adaptive weight coefficients. Due to the inevitable noise of gene data, the BM3D algorithm is used to denoise the decomposed reflection image [13]. To deal with the phenomenon that the denoised image becomes smooth and the image details are lost, the MSDB algorithm is used to enhance the image details at the same time as denoising [14]. In the enhanced network, the decomposed illumination map is input into the network for training [15]. The enhancement network part adopts the design of adding channel attention network layer U-Net network structure, which can transfer the feature map in the up and downsampling of the image so that the upsampling network can judge the lost pixels according to the transferred feature map, suppress the occurrence of a blur, and obtain high-quality light map [16]. The channel attention network layer added in the network obtains the gene image information that needs attention through learning and obtains the relationship between gene image channels [17]. BM3D and MSDB algorithms denoise and enhance the image details of gene reflection maps, eliminate the influence of reflection map noise, and improve the image quality when reconstructing the image [18].

## 3. Results and Analysis

**Figure 1:**
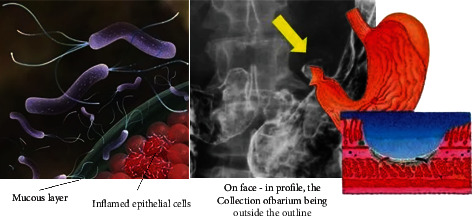
Map of pathogenesis of peptic ulcer.

As we all know, the occurrence of peptic ulcer is caused by the imbalance between the attacking factors that have a damaging effect on the gastroduodenal mucosa and the protective and repairing factors of the mucosa itself. When the damage factor is greater than the defense factor, the occurrence of PU is promoted as shown in Figure 1.

### 3.1. Image Enhancement Site Recognition and Peptic Ulcer Analysis

Peptic ulcer disease (PUD) is an inflammatory defect that occurs in the gastrointestinal mucosa, most commonly in the stomach and duodenum, and is a common global disease with a prevalence of about 5–10% in the general population and the annual incidence rate of 0.1–0.3%. The incidence of peptic ulcer disease has declined significantly in recent decades. However, the incidence of children's digestive system diseases is increasing year by year, and its causes are complex and diverse.

**Figure 2:**
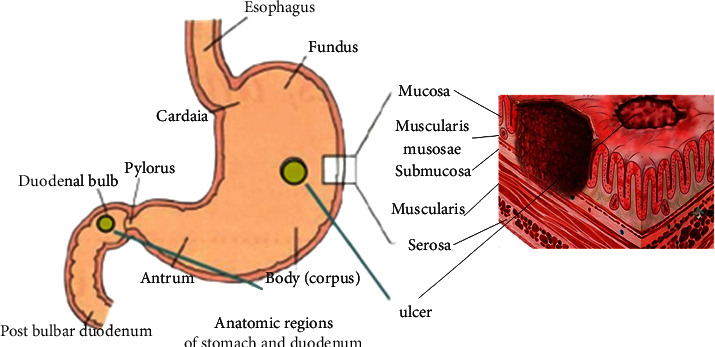
Molecular mechanisms of the development of peptic ulcer.

The history of chronic gastric and duodenal inflammation is often atypical, and the symptoms are nonspecific. Moreover, due to the operational difficulties in the collection and analysis of gastric juice in children, the changes in gastric acidity during childhood are unstable, so it is difficult to judge. Endoscopy has become the best method for diagnosing ulcer disease, assessing the degree of ulcer activity, determining the presence or absence of Hp infection and evaluating curative effect. Therefore, it is necessary to increase the recognition efficiency of gastroscopic images through image enhancement algorithms and to perform clinical classification and diagnosis of children with gastric ulcers as soon as possible for individualized treatment or nursing care to improve the prognosis of patients. The location of peptic ulcer can be shown in Figure 2.

As shown in Figure 2, peptic ulcer is a relatively common disease among digestive system diseases. It has a long course of disease, slow onset, and extremely high recurrence rate. It is difficult to cure the disease completely, and it also seriously affects people's work and life.

There are many causes for onset of peptic ulcer in children, and the following are the most common factors:

1. Genetic factors: the study found that relatives of patients with chronic peptic ulcer were twice as likely to develop ulcer disease, while immediate family members of patients with peptic ulcer disease were more likely to develop symptoms such as stomach ulcers. According to incomplete statistics, one-third of children with peptic ulcer disease have a significant family history.
2. Improper diet and bad living habits: the child itself is in the development stage, and the resistance of various parts of the body is insufficient. In addition, children are prone to overeating and eating some spicy and other irritating foods without adult care. These are easy to induce gastritis, which can form stomach ulcers. At the same time, children can easily induce ulcers in an environment of passive smoking.
3. Psychological factors: medical workers have found in studies that when people are excited, the gastric mucosa is hyperemic, secretion is increased, and gastric motility is enhanced; when the gastric mucosa is pale, secretions are reduced, and gastric motility is weakened when the mood is low. Studies have also confirmed that the secretion of gastric juice is influenced by emotional and biofeedback control.

**Figure 3:**
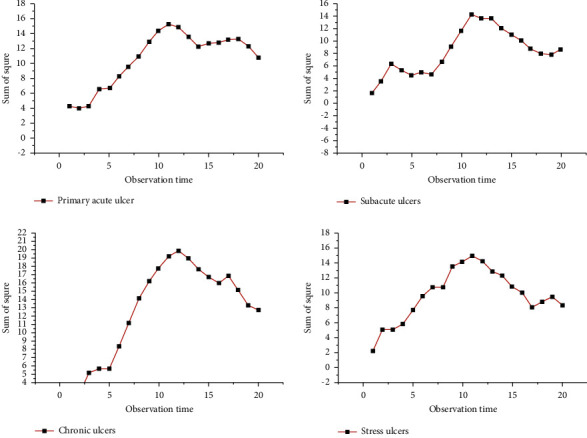
The final enhancement result of the side-scan sonar image.

The disease is classified according to Tudor's clinical classification of peptic ulcer: ① gastric ulcer; ② duodenal ulcer; ③ complex ulcer: gastric and duodenal ulcer coexist. Clinical classification of peptic ulcer based on Tudor.  Figure 3 shows the final enhancement results of gastroscopic images of four kinds of ulcers.

Tables 1 and 2 are data tables for gastric ulcer detection rates in children and data tables for duodenal ulcer detection rates, respectively.

**Figure 4:**
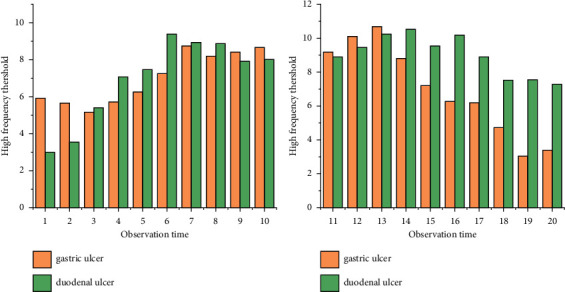
The effect of image enhancement algorithm after dark light gene enhancement.

As shown in Figure 4, the gastroscopic detection rates of gastric ulcers and duodenal ulcers in children, respectively, decreased with the increase of time. Gastroscopic detection rates of duodenal ulcers gradually stabilized.

### 3.2. Endoscopic Manifestations in Patients with Peptic Ulcers

Endoscopic manifestations in patients with peptic ulcer are shown in Table 3.

**Figure 5:**
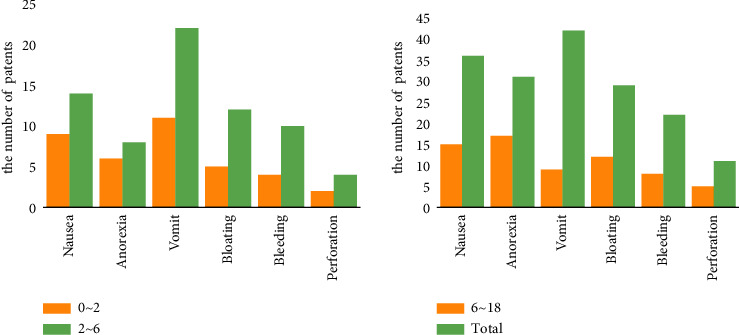
Clinical manifestations in patients of different ages.

The clinical presentation of patients of different ages also varied, and the results showed that their population distribution was remarkable, bleeding, bloating, vomit, anorexia, and nausea, as shown in Table 4 and Figure 5.

The clinical manifestations of peptic ulcer in children are mainly regular abdominal pain, the timing of gastroscopy in cases is mainly active period, and in the case-control study of factors related to PU occurrence, the results of univariate analysis show that the factors related to PU incidence include male, primary school education, manual labor occupation, irregular life, smoking, drinking, lack of sleep, irregular diet, consumption of milk, consumption of coffee, consumption of fried foods, and history of cardiovascular disease. The results of multifactor analysis showed that irregular life, lack of sleep, irregular diet, consumption of fried foods, history of cardiovascular disease were the main factors related to the occurrence of PU, and consumption of milk was negatively correlated with the occurrence of PU.

### 3.3. Analysis of the Detection Rate of Peptic Ulcer in Children of Different Age Groups

**Figure 6:**
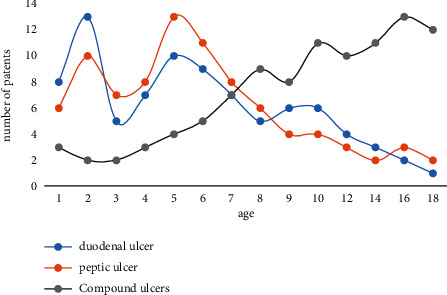
Plot of peptic ulcer detection results.

According to the data table of children's peptic ulcer detection rates recorded in Tables 1 and 2, a comparative map of children's peptic ulcer detection rates can be obtained.  Figure 6 shows a comparative plot of detection rates of peptic ulcers in children.

**Figure 7:**
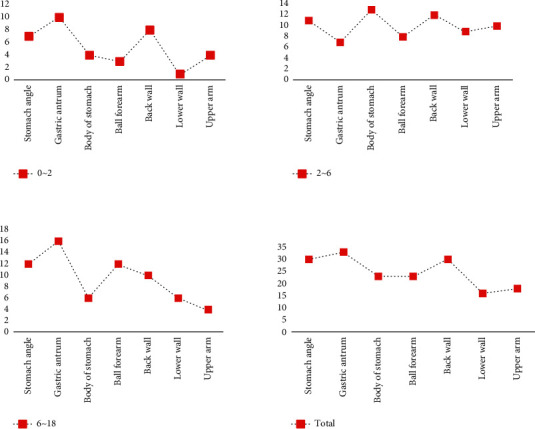
Map of peptic ulcer manifestations at different ages.

Through medical endoscopy, the pathological changes of various organs in the human digestive tract can be clearly seen, and various abnormal manifestations in the organs can be found, so as to help medical staff better formulate follow-up physical examination and medical treatment plans. Based on the data in Table 3, a comparative plot of endoscopic performance in children with peptic ulcer can be plotted, as shown in Figure 7.

From Figure 7, it can be seen that among the endoscopic manifestations of gastric ulcer in children, gastric ulcer is the most obvious in children aged 2 to 6 years, with 13 cases of gastric ulcer; in children aged 6 to 18 years, the endoscopic manifestation of gastric ulcer is the most obvious, and 16 patients have gastric ulcer. In the endoscopic manifestations of duodenal ulcers in children, the number of posterior wall ulcers is generally the highest, reaching 30.

**Figure 8:**
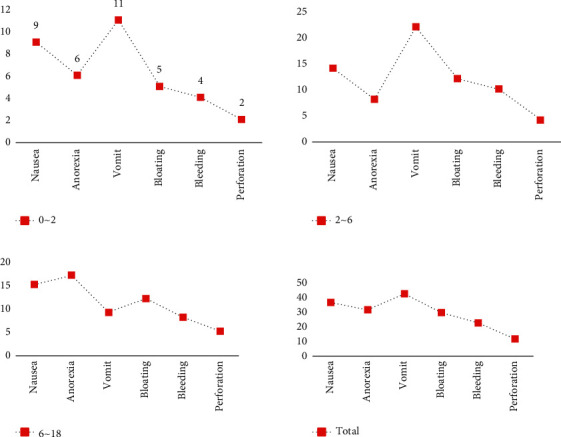
Different clinical manifestations of peptic ulcers.

Peptic ulcer is a disease with complex etiology, which is generally manifested by epigastric pain, acid reflux, belching, nausea, vomiting, etc., and in severe cases, there may be complications such as gastrointestinal bleeding, perforation, pyloric obstruction, and cancer. As children age, the function of the neuroendocrine system changes accordingly, which leads to gastrointestinal dysfunction and pathological changes leading to different types of peptic ulcers. Based on the statistics in Table 4, statistical charts of the clinical manifestations of peptic ulcers at different ages can be plotted, as shown in Figure 8.

As can be seen from Figure 8, when children suffer from peptic ulcer disease, the main clinical symptoms are perforation, bleeding, bloating, vomiting, anorexia, and nausea, and the degree of clinical symptoms in different ages is also different.

## 4. Discussion

In this study, the three clinical symptoms of nausea, anorexia, and vomiting were most obvious in preschool children with peptic ulcer disease. School-age children with peptic ulcer disease are usually accompanied by symptoms such as nausea and vomiting. This experiment shows that the gastroscopic results of peptic ulcer in children show different characteristics with different ages.

Peptic ulcer is a global frequently occurring and common disease. The main clinical manifestation is chronic epigastric pain, which is characterized by chronic, periodic, and rhythmic pain. Acid-suppressing drugs and alkaline drugs can often relieve pain. Complications are more commonly bleeding, perforation, and pyloric obstruction, and about 5% of gastric ulcers can also develop cancer. The incidence is higher in men than in women, and can occur at any age, with the most common age group being 20–60 years. A variety of factors can affect the occurrence and development of ulcer disease. With the acceleration of social development, people's mental stress increases, and the normal life is lost. The study found some unhealthy behaviors, unreasonable dietary structure, mental and psychological factors, and pyloric spiral. Bacillus is an important risk factor for peptic ulcer [18]. The purpose of ulcer disease treatment is not simply to make the symptoms disappear, but more importantly, to achieve mucosal recovery, prevent recurrence, prevent ulcer malignancy, and improve the quality of life and survival rate of patients.

Although the pathogenesis and etiology of peptic ulcer are different, the clinical manifestations are basically similar [19]. It is generally believed that the occurrence and development of peptic ulcer is due to the imbalance between the defense factors and damage factors of the gastric and duodenal mucosa. The gastric mucosal damage factors refer to (1) gastric acid and pepsin, (2) drug factors such as NSAIDs, (3) Hp infection, (4) bile salts, and (5) ethanol. Defense factors are (1) gastric mucosal mucus barrier, (2) mucosal blood flow, (3) prostaglandins and epidermal dust growth factor, (4) cell regeneration, and (5) bicarbonate and so on. When the defense factor to the gastric mucosa is less than the damage factor, peptic ulcer may form. In addition, there are other factors such as mental factors, diet, smoking, and genetic factors, which constitute the complex pathogenic mechanism of ulcer occurrence and development. [20]. In addition, gastric ulcer and duodenal ulcer have different pathogenic mechanisms. The former is mainly due to the weakening of defense and repair factors, while the latter is the enhancement of damage factors, or both.

## 5. Conclusions

This paper confirms that gastroscopy can effectively diagnose peptic ulcer disease in children, but the accuracy of diagnosis at different ages is yet to be studied. Therefore, in the future, it is still necessary to deeply reveal the mechanism of action of peptic ulcers, and clarify clinical typing by designing a variety of identification protocols and reasonable data training methods. In addition, the causes of pediatric peptic ulcers involve a variety of influencing factors, which may make them of great research value in the study of other digestive diseases in children. At present, there are still some problems, such as the image brightness cannot be self-adaptive control, only by learning the illumination factor to enhance the image; it depends on the extension of the data set; The fuzzy problems need to be solved, and the future work will study the above problems. Therefore, in the future, it is still necessary to reveal the mechanism of metformin on the ad, and design individualized medication schemes and reasonable clinical trials to determine the long-term prognosis. In addition, metformin action sites are involved in the cross pathways of many diseases, which may make it more valuable in the study of the mechanism and clinical treatment of many diseases. For this reason, the patient did not recognize the diversity of his condition during the treatment process, and these causes can be overcome in daily life, which can avoid the recurrence of ulcers. Therefore, the rapid identification and diagnosis of various peptic ulcers can serve as a warning to the relevant population.

## Figures and Tables

**Table 1:** Detection rate of gastric ulcer.

Age	Number of peopleinspected	Ulcer/case	Ulcer detectionrate (%)
0 ∼ 2	264	21	8.0
2 ∼ 6	365	31	8.5
6 ∼ 18	321	34	10.6
Total	950	86	9.0

**Table 2:** Detection rate of duodenal ulcer.

Age	Number of peopleinspected	Ulcer/case	Ulcer detectionrate (%)
0 ∼ 2	209	16	7.7
2 ∼ 6	363	39	10.7
6 ∼ 18	402	32	8.0
Total	974	87	8.8

**Table 3:** Endoscopic manifestations of patients with gastric and duodenal ulcer.

Age	Gastric ulcer			Duodenal ulcer			
	Stomach angle	Gastric antrum	Body of stomach	Ball forearm	Back wall	Lower wall	Upper arm
0 ∼ 2	7	10	4	3	8	1	4
2 ∼ 6	11	7	13	8	12	9	10
6 ∼ 18	12	16	6	12	10	6	4
Total	30	33	23	23	30	16	18

**Table 4:** Clinical manifestations of children with peptic ulcer.

Age	Nausea	Anorexia	Vomit	Bloating	Bleeding	Perforation
0 ∼ 2	9	6	11	5	4	2
2 ∼ 6	14	8	22	12	10	4
6 ∼ 18	15	17	9	12	8	5
Total	36	31	42	29	22	11

## Data Availability

The authors do not have permission to share data from the data provider.
